# A “Boil” Being the Clue to Think beyond Typical Bacterial Pathogens in Community-Acquired Pneumonia

**DOI:** 10.1155/2022/8984170

**Published:** 2022-03-29

**Authors:** Jeanna Auriemma

**Affiliations:** Wake Forest School of Medicine, Department of Pediatrics, 1 Medical Center Boulevard, Winston-Salem, NC 27157, USA

## Abstract

Empyema necessitans is an exceptionally rare complication of bacterial pneumonia in the pediatric population. It occurs when the infection extends from the lung parenchyma to the chest wall by forming a fistula, which leads to infection of the surrounding soft tissue. In this case, a 13-year-old boy is found to have empyema necessitans caused by *Actinomyces meyeri*, with a preceding clue to the diagnosis being that he was treated for a superficial chest wall abscess several weeks prior to developing significant respiratory symptoms. Providers should be aware of this entity as it requires obtaining cultures to identify the appropriate pathogen and avoid treatment failure as it has implications for antibiotic choice and length of therapy.

## 1. Introduction

Community acquired pneumonia is the most common cause of death in children globally and up to 155 million cases occur annually [1, 2]. The most common cause of bacterial pneumonia in children is *Streptococcus pneumoniae*. Pulmonary complications of pneumonia may include lung abscess, necrotizing pneumonia, parapneumonic effusion/empyema, pneumothorax, and pneumatoceles. Most cases of pediatric pneumonia are uncomplicated, and complications of pneumonia are seen more often in adults than in children.

## 2. Case Presentation

A 13-year-old young man presents to the emergency department with cough, fever, and chest pain. Four weeks previously, he had an acute onset of nonproductive cough and subjective fever. He was diagnosed clinically with pneumonia and treated as an outpatient. He completed a 7-day course of oral levofloxacin. His cough persisted after treatment, but he was not having any further fevers. Two days ago, he developed new fever and left lateral chest pain.

He currently reports pain with deep respirations that fails to improve with acetaminophen. His appetite has decreased, and he has lost 10 pounds over the past month.

He has no long-term medical problems and has had no previous hospitalizations or surgeries. Two months ago, he had a superficial abscess over his left chest wall which was incised and drained by his primary care physician. He took 10 days of clindamycin, and his skin has healed completely since that time. A wound culture from the drained purulence only grew normal skin flora. He denies previous skin and soft tissue infections, and there is no history of methicillin-resistant *Staphylococcus aureus* (MRSA) among household contacts. He has had no foreign travel or unusual environmental or animal exposures. His review of systems is otherwise negative.

In the emergency department, he is afebrile with a normal heart rate and blood pressure. He is breathing between 14 and 20 times per minute and has oxygen saturations over 95% on room air.

On examination, he is nontoxic in appearance. He is breathing comfortably without use of accessory muscles. Lung auscultation reveals greatly diminished breath sounds throughout the left lung with no adventitious sounds. He has egophany when performed over the left lung. His right lung examination is normal. He has no skin lesions, nasal congestion, dental caries or other evidence of poor dental hygiene, or other pertinent findings.

Labs are significant for a white blood cell count of 16,100/mm^3^ with a neutrophilic predominance and mild normocytic anemia of 11.6 g/dL. A basic metabolic panel including electrolytes and renal function is unremarkable. C-reactive-protein (CRP) is 73.6 mg/L. A blood culture is obtained which ultimately returned negative.

**Figure 1:**
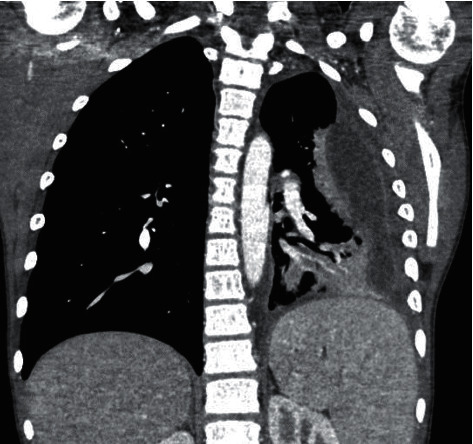
Volume loss noted in the left lung with large loculated fluid collection in the left pleural space.

His chest radiograph shows a loculated pleural effusion in the left hemithorax concerning for empyema. A chest CT reveals the fluid collection has peripheral rim enhancement with a bi-lobed appearance, with the superior lobe measuring 7 cm in axial dimension and the inferior lobe measuring 8.6 cm. The total length is 12.5 cm in the craniocaudal dimension (Figure 1). There is an associated area of consolidation in the left lower lobe with air bronchograms consistent with pneumonia as well as reactive mediastinal lymphadenopathy.

He is empirically started on intravenous (IV) vancomycin and ceftriaxone for presumed complicated community-acquired pneumonia. Due to the size of the presumed empyema, he is taken to the operating room for chest tube placement. The empyema is successfully drained with 250 mL of foul smelling pus removed from his left chest and a bacterial culture is obtained. The culture became positive revealing the diagnosis, and his antibiotics are adjusted accordingly.

## 3. Final Diagnosis

The culture obtained from the empyema fluid grew 3+ *Actinomyces meyeri*, revealing his diagnosis of pulmonary actinomyces.

## 4. Hospital Course

Once his culture was positive and susceptibilities were obtained, his antibiotics were adjusted and he was placed on IV ampicillin monotherapy. He received fibrinolytic therapy with tissue plasminogen activator (TPA) through his chest tube every 24 hours for 3 days. He remained on room air throughout his stay. His chest tube was removed after 8 days when his tube output was minimal, and he was sent home with a peripherally inserted central catheter to complete a total of 4 weeks of ampicillin. CRP obtained at discharge for trending purposes was 22.5 mg/L. At the completion of his IV ampicillin course, his CRP normalized to 0.5 mg/L and he was transitioned to oral amoxicillin for 6 months.

## 5. Discussion

Empyema necessitans is a rare complication of empyema. It occurs when the infection extends from the thorax outward to the chest wall by forming a fistula, leading to infection in the nearby soft tissue. In this patient, what was thought to be an isolated superficial soft tissue abscess was more likely to be an extension of his underlying indolent pneumonia. Actinomyces are fastidious, predominately anerobic Gram-positive bacilli that are challenging to culture unless under ideal conditions [3] and incubation can take up to 14 days [4]. The location of the abscess on the left chest wall directly over his area of most profound lung involvement was the clue to this. Empyema necessitans has been best described in the literature in association with *Mycobacterium tuberculosis* and *Actinomyces israelii*. It has also been documented in infections with *Staphylococcus*, *Escherichia coli*, *Pseudomonas*, *Klebsiella*, *Proteus*, *Aspergillus*, streptococcus, and anaerobes [5–7].

In children, empyema is treated with drainage of the fluid although there is controversy over optimal treatment practices. In general, drainage can be achieved with chest tube placement with the addition of fibrinolytics or video-assisted thoracoscopic surgery (VATS) and similar outcomes are expected. VATS is most typically reserved for patients who have failed chest tube with fibrinolytics [8].

Actinomyces species are considered commensal organisms in the oropharynx, and pulmonary actinomycosis likely results from aspiration events [9]. It is an exceptionally rare entity in children and adolescents and, thus, there is not a typical presentation or framework for work-up. The population affected tends to be middle-aged males particularly those with structural lung diseases [10]. *Actinomyces meyeri* has a high propensity for hematogenous spread to other organs including long bones, liver, muscle, and brain compared to other *Actinomyces* species. This has important implications on work-up based on the exam and symptoms present [11]. The recommended length of therapy is typically IV penicillin for several weeks, followed by 6 to 12 months of oral penicillin or amoxicillin [9].

## 6. Conclusion

Medical providers should consider a recent history of skin and soft tissue infection on the chest wall in patients with pneumonia as a clue to a potentially rare causative organism, which may have implications for targeted antibiotic therapy and length of treatment.

## Data Availability

As a case report, no underlying data were used in the preparation of this manuscript.
